# Development of myocardial edema following acute bouts of intense physical exertion in healthy active men: a Cardiovascular Magnetic Resonance (CMR) study

**DOI:** 10.1186/1532-429X-13-S1-O111

**Published:** 2011-02-02

**Authors:** Myra S Cocker, Mark J Haykowsky, Matthias G Friedrich

**Affiliations:** 1 Stephenson Cardiovascular Magnetic Resonance Centre, Libin Cardiovascular Institute of Alberta, Departments of Cardiac Sciences and Radiology, University of Calgary., Calgary, AB, Canada; 2 Faculty of Rehabilitation Medicine, University of Alberta, Edmonton, AB, Canada

## Background

Serological markers of cardiac injury are elevated following intense exertion. Whether myocardial tissue is actually injured remains unresolved, although we have found evidence for systolic and diastolic dysfunction following exertion. Furthermore, we have also observed that a significant proportion of well-trained elite endurance athletes present with evidence of myocardial fibrosis at rest.

Myocardial edema reflects an acute reversible injury that may be associated with reduced ventricular compliance. Accordingly, the purpose of the current investigation was to determine whether myocardial edema develops acutely in active men challenged with an incremental test to exhaustion and a high-intensity interval exercise training session, as visualized by T2-weighted imaging.

## Methods

16 physically active men (age 33±11 years) were recruited for a 2-day study. LV function and T2-weighted imaging were performed on a 1.5T MRI system, prior to and within an hour of exertion. During study day 1, participants completed an incremental test to exhaustion on a braked cycle ergometer, where load was increased by 15-25 Watts every 2 minutes until physical exhaustion. The point of exhaustion was considered to be the maximal power output. During study day 2, a high-intensity interval training session was performed where athletes completed 15 repetitions of cycling at maximal power output for 1 minute followed by 2 minutes of light recovery.

The extent of myocardial edema was assessed quantitatively using semi-automated detection, where myocardial regions that had signal intensity (SI) above a threshold of twice the mean SI of skeletal muscle were considered to be edematous. Global edema was measured by normalizing myocardial SI to skeletal muscle SI, generating a T2-ratio.

## Results

The ratio of global myocardial edema increased following both incremental exertion to exhaustion (1.72±0.20 pre-exertion vs. 1.84±0.26 post-exertion, p=0.03) and intense interval training (1.71±0.14 pre-exertion vs. 1.92±0.14 post-exertion, p<0.001).

**Figure 1:**
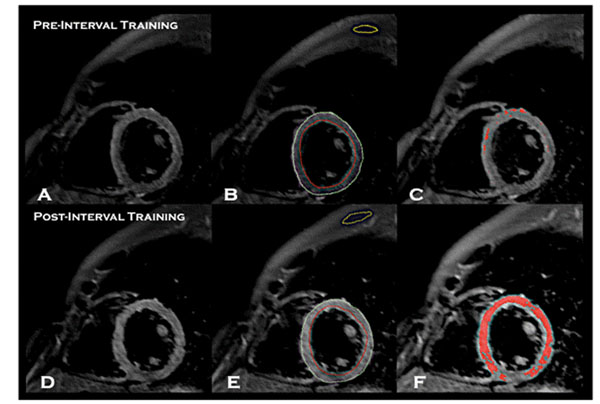
Visibly increased extent of myocardial edema in an endurance athlete following high-intensity interval training using T2-weighted cardiac MRI imaging (panel D vs. panel A). Extent of myocardial edema was quantified by tracing endocardial (red contours) and epicardial contors (green contours) to determine myocardial signal intensity, as well as yellow contours to measure skeletal muscle signal intensity (panels B, E). Mean signal intensity of skeletal muscle was multiplied by a factor of 2, and this number was utilized as a threshold to semi-automatically detect the extent of myocardial edema (red overlay, panels C and F). In this example, the extent of myocardial edema increased from 1.5% to 59.6% of LV mass following intense interval training.

The spatial extent of myocardial edema also increased following both incremental exhaustion challenge (17.9±17.9 pre-exertion vs. 32.1±24.5 post-exertion, p=0.003) and high-intensity interval training (18.1±16.1 pre-exertion vs. 39.4±16.6 post-exertion, p<0.001) (Figure 1). The spatial extent of edema after intense interval exercise was inversely related to LVEDV (r=-0.610, p=0.021) and LVESV (r=-0.739, p=0.003), and positively related to LVEF (r=0.706, p=0.005).

## Conclusion

We provide first evidence for the development of myocardial edema following an acute incremental challenge to exhaustion and high-intensity interval exertion in healthy men. The extent of edema may be related to diastolic dysfunction. These findings have implications for the safety of sport for athletic and non-athletic populations.

